# Salivary Neutrophil-to-Lymphocyte Ratio as a Prognostic Predictor of Oral Premalignant and Malignant Disorders: A Prospective Study

**DOI:** 10.7759/cureus.56273

**Published:** 2024-03-16

**Authors:** Dilip B Magdum, Noopur A Kulkarni, Pratibha G Kavle, Swati Paraye, Pritam S Pohankar, Amol V Giram

**Affiliations:** 1 Department of Oral Pathology and Microbiology, Bharati Vidyapeeth Dental College and Hospital, Sangli, IND; 2 Department of Oral Pathology and Microbiology, Pandit Deendayal Upadhyay Dental College, Solapur, IND; 3 Department of Oral Pathology and Microbiology, Bharati Vidyapeeth Dental College and Hospital, Navi Mumbai, IND; 4 Department of Oral Medicine and Radiology, Saraswati Dhanwantari Dental College and Hospital, Parbhani, IND

**Keywords:** saliva, prognosis, lymphocyte, neutrophil, oral cancers

## Abstract

Introduction: Inflammation is a definitive characteristic of carcinogenesis. The neutrophil-to-lymphocyte ratio (NLR) is an easy and efficient indicator of inflammation and a valuable marker in individuals with malignancies. The present study was performed to ascertain NLR values in salivary samples collected from individuals with oral premalignant disorders (OPMDs) and to assess the prognostic significance of NLR in distinguishing OPMDs from oral malignancies.

Materials and methods: This study was conducted on 50 patients histopathologically diagnosed with OPMDs with mild dysplasia. The patients were provided with standard medicinal treatment, encouraged to quit their habits, and followed up for one year at three-month regular intervals. During the follow-up, 29 (67.4%) patients completely recovered, whereas 14 (32.6%) developed oral malignancies. Salivary samples were collected at baseline (T0) and one-year follow-up (T1). The total salivary neutrophils and lymphocytes were counted using an improved cell counting method with a Neubauer chamber. The NLR values were calculated at T0 and T1. The paired t-test was used to compare the NLR values at T0 and T1. The cutoff value of the NLR was determined using the receiver operating characteristic (ROC) curve. The Youden index was used to determine the optimal cutoff NLR values in the groups. Statistical significance was set at p ≤0.05.

Results: OPMDs were predominantly observed in males, with leukoplakia being the most prevailing one. Erythroplakia exhibited the highest propensity for malignant transformation, and habitual consumption of alcohol and tobacco was identified as a risk factor for this transformation. NLR increased in both premalignant and malignant conditions. NLR value equal to or exceeding 4 was determined to be a reliable indicator for the occurrence of oral cancer in patients with OPMDs. The ROC curve analysis yielded a sensitivity and specificity of 92%, with an area under the curve (AUC) of 0.928.

Conclusion: The poor prognosis of oral cancers was associated with higher NLR values. NLR values in salivary samples can serve as an independent reliable predictor in oral cancer and OPMDs.

## Introduction

Oral squamous cell carcinoma (OSCC) is a prevalent neoplasm of the craniofacial region and is primarily localized within the oral cavity, accounting for 90% of all cases [1]. Most instances of oral cancer are linked to behaviors, such as tobacco and areca nut consumption. These instances are preceded by asymptomatic clinical lesions commonly known as oral premalignant disorders (OPMDs). OPMDs commonly found in Asian countries, such as India, include leukoplakia, erythroplakia, erosive lichen planus, and oral submucous fibrosis (OSMF) [2]. One of the initial epidemiological studies that evaluated the probability of OPMDs in India documented that 80% of oral cancers were heralded by OPMDs [3]. Despite significant progress in the therapeutic management of advanced OSCC, there has been limited improvement in the overall survival rate of individuals with oral cancer [4]. Prognostic biomarkers are of paramount significance in the context of treatment strategies as they provide indispensable insights into the holistic prognosis of patients. However, the utilization of molecular biomarkers that necessitate the use of tissue specimens for analysis places a significant burden on patients as it mandates the adoption of an invasive methodology for the collection of samples [5]. In addition, the time required for the analysis of tissue biomarkers is extended. Consequently, there is an imperative requirement for the development of convenient and noninvasive prognostic biomarkers for oral cancer. Prompt diagnosis is of utmost importance in managing the potential malignant progression of OPMDs and enhancing the overall survival of patients.

It is widely recognized that inflammation and cancer are strongly correlated [6]. The development of tumors, including their initiation, progression, and metastasis, is influenced by inflammatory responses [7]. Several investigations have suggested that the prognosis of different types of cancer is associated with the quantities of lymphocytes, monocytes, neutrophils, and platelets present in the bloodstream [8,9]. These indicators can be influenced by oxidative stress, chemokines, and cytokines during cancer onset and progression.

The neutrophil-to-lymphocyte ratio (NLR) is an important biomarker that has been documented as a prognostic indicator in oral cancer [8,9]. However, contradictory findings have been reported in previous studies [10-12]. Some studies have reported NLR as a positive predictor of oral cancers [10,11], whereas other studies have reported NLR as a negative predictor [7,12]. Moreover, all of these studies were conducted in histopathologically diagnosed cases of OSCC and in the blood samples of the patients. Saliva-based diagnostics have been reported to exhibit a considerable level of sensitivity (≥95%) and specificity, thereby demonstrating the test's ability to accurately discern positive or negative cases [13]. Therefore, as there has been no previous investigation into the evaluation of NLR in salivary samples of individuals with OPMDs, the current study was undertaken to ascertain the prognostic significance of NLR in salivary samples obtained from patients with OPMDs who subsequently developed OSCC within a one-year follow-up period.

## Materials and methods

Study design

A non-randomized, prospective cohort study spanning from August 2022 to December 2023 was undertaken in the Department of Oral Medicine in collaboration with the Department of Oral Pathology at the Saraswati Dhanwantri Dental College Parbhani. Ethical approval was obtained for the study from the Institutional Ethical Committee (EC/NEW/INST/2022/3986/F21), and written informed consent was obtained from all patients after explaining the study protocol. The study was conducted following the Standard Protocol Items: Recommendations for Interventional Trials (SPIRIT) guidelines.

Sample size estimation

The sample size was calculated using the G Power software (Ver. 3.1 Franz Faul, Universität Kiel, Germany). Power analysis revealed that a sample size of 42 patients would provide a power of 80% (1-β) with an alpha value of 5% and an effect size of 0.8, as derived from a previous study [14]. Therefore, considering the 10% loss to follow-up, 50 patients were included in this study.

Patient participation and eligibility criteria

**Figure 1:**
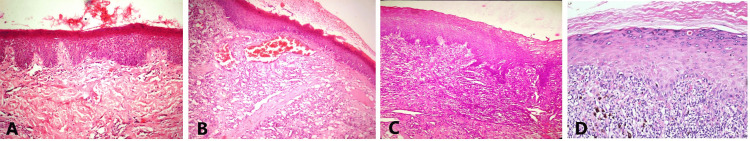
Histopathological presentation of oral premalignant disorders with mild dysplasia: A. Erythroplakia, B. oral submucous fibrosis, C. leukoplakia, D. lichen planus

The inclusion criteria were as follows: histopathologically diagnosed untreated cases of OPMDs, such as leukoplakia, erythroplakia, lichen planus, and OSMF showing mild dysplastic changes according to the World Health Organization (WHO) classification 2017 [15] (Figure 1).

Patients who had factors that may potentially influence the NLR, including acute and chronic infections, oral inflammation apart from the presence of OPMDs, periodontally compromised patients, prior radiotherapy, presence of oral cancer, salivary gland tumors, chronic renal insufficiency, long-standing inflammatory disorders, cardiovascular and liver problems, chronic hematological ailments, and recent administration of steroids or immunosuppressive agents, were excluded from the study. All patients were administered standard medicinal remedies based on the type of OPMDs [16]. Details regarding the demographic data and history of habits were obtained for all patients. All patients were instructed and motivated to stop habits, such as alcohol consumption, tobacco (both smoking and smokeless), and spicy foods. All patients received thorough oral prophylaxis one week before taking oral salivary samples.

Saliva collection

Patients were instructed to abstain from consuming any food or beverages for a minimum duration of 30 min and to refrain from spitting before providing oral salivary samples. This precautionary measure was taken to prevent the removal of neutrophils and lymphocytes from the oral cavity, thus ensuring the accuracy of the total neutrophil count and avoiding a potential bias. Neutrophils and lymphocytes were assessed between noon and 2 pm to eliminate any variations caused by the natural daily rhythm of migration of these cells within the oral cavity. Unstimulated saliva, in its entirety, was procured from the floor of the mouth of all the patients. The rinse samples were vortexed for 1 min and then centrifuged at 4 °C using a Multi FC5706 230V (OHAUS Corporation, Mumbai, India) at a relative centrifugal force (RCF) of 500 for 10 min. The supernatant was discarded, and the resulting pellet was resuspended in 2 mL of PBS. To ensure proper filtration, the resuspended pellet was passed through nylon meshes with pore sizes of 31.5 μm and 10.0 μm (Labcare Scientific, Coimbatore, India). The filtered fraction was then subjected to centrifugation at 4 °C with an RCF of 500 for 10 min. The remaining pellet was resuspended in supplemented PBS (Thermo Fisher Scientific, Mumbai, India) containing 1.5 mmol·L^−1^ CaCl_2_ and 1 mmol·L^−1^ MgCl_2_-H_2_O (sPBS).

Neutrophil and lymphocyte count determination

Following this, 20 mL of the suspension was stained with 10 mL of Turks’ solution for 10 min. Neutrophil and lymphocyte counts were enumerated using an improved Neubauer chamber (NCC-3071, Ravi Scientific Industries, Delhi, India) using an improved cell counting method with a Neubauer chamber by Zhang et al. [17]. NLR was computed by dividing the absolute neutrophil count by the absolute lymphocyte count.

After the collection of oral samples (T0), each selected patient was administered standard medicinal treatment for OPMDs with mild dysplasia and was recalled every three months to evaluate the progress of the lesion. After an average of one year of follow-up, a second oral rinse sample was collected and all clinical parameters of the study were recorded (T1). All the patients underwent a second biopsy for histopathological confirmation of the progress of the lesion. The histopathological parameters such as hyperchromatic nuclei, pleomorphism, abnormal mitosis, interrupted basement membrane, and epithelial islands in the stroma were considered malignant changes.

Statistical analysis

Data were analyzed using SPSS version 22.0 (SPSS, Inc., Chicago, IL). Qualitative variables are presented as frequencies, and quantitative variables are presented as means and standard deviations. The paired t-test was used to compare the NLR values at T0 and T1. The cutoff value of NLR was determined using the receiver operating characteristic (ROC) curve. The Youden index was used to determine the optimal cutoff NLR values for each group. Statistical significance was set at p ≤0.05.

## Results

**Figure 2:**
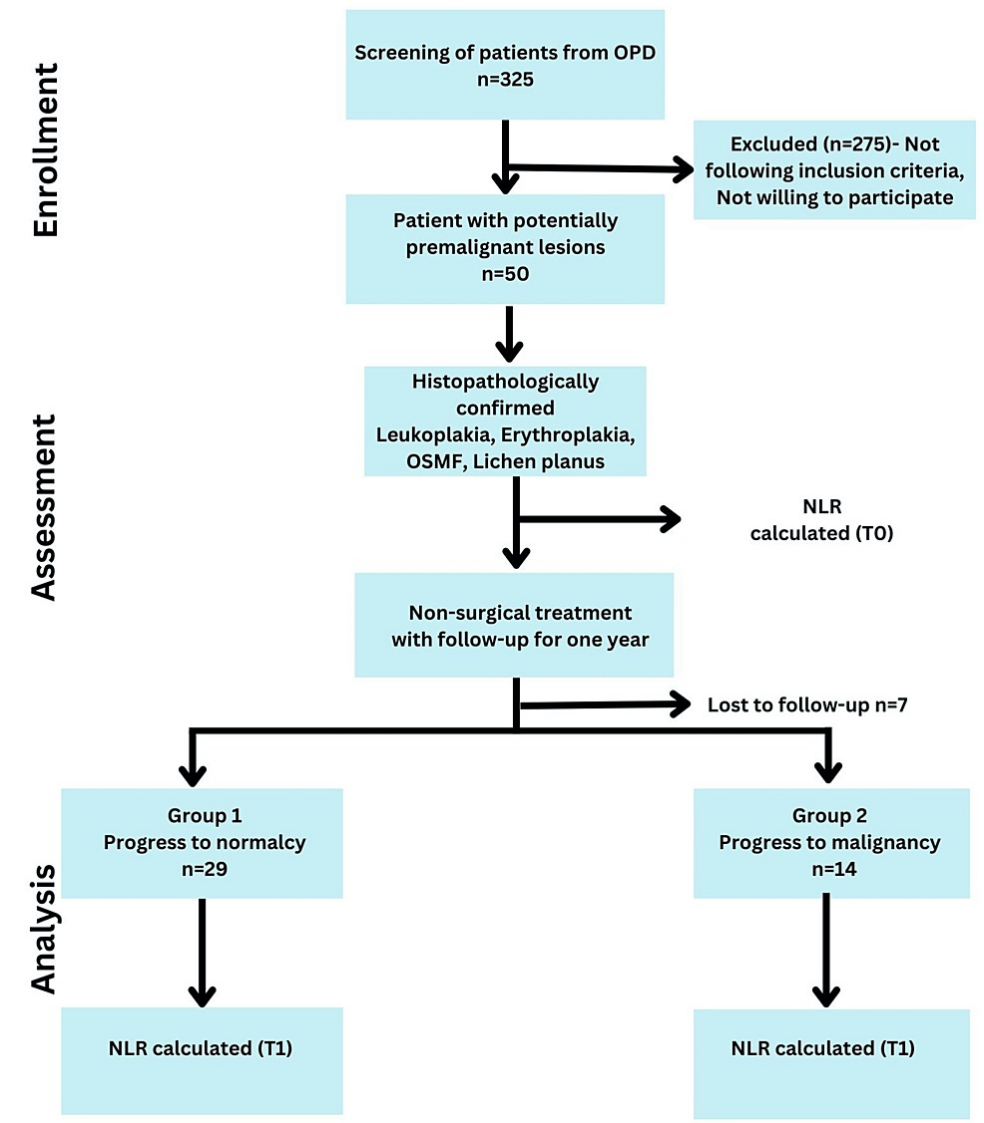
Study design OPD: outpatient department; NLR: neutrophil-to-lymphocyte ratio; OSMF: oral submucous fibrosis; T0: baseline; T1: at a follow-up period of one year.

A total of 325 patients from the Outpatient Department (OPD) of Oral Medicine were screened. Fifty patients fulfilled the eligibility criteria, and seven were lost during the one-year follow-up period. Therefore, a total of 43 patients were included in the analysis. Of the 43 patients with OPMDs, 29 (67.4%) showed complete remission of the lesion (group 1), and 14 (32.6%) patients showed progression of lesions to malignancy (group 2) during the follow-up period (Figure 2). 

Descriptive analysis of the data revealed no statistically significant sex differences, and most of the patients were males (16 (37.2%) in group 1 and 8 (18.6%) in group 2). The mean age of the patients in group 1 was 33.65±3.75 years, whereas in group 2, it was 34.64±5.82 years with no statistically significant differences. The patients in group 1 were mostly tobacco users (smoking + smokeless tobacco) for a shorter duration of time (2.00±2.44 years for smoking and 3.62±3.04 years for smokeless tobacco), whereas in group 2, they were consuming both tobacco and alcohol for a longer duration of time (>4 years). Seventeen OPMDs (39.5%) present in the buccal mucosa showed complete remission, whereas nine OPMDs (13.9%) present on the floor of the mouth showed malignant transformation. In group 1, 17 (39.5%) OPMDs were found mainly on the left side, compared to 11 (25.5%) OPMDs on the right side in group 2. The most common type of OPMD was leukoplakia 21 cases (48.7%), followed by erythroplakia 12 cases (27.8%), OSMF five cases (11.6%), and lichen planus five cases (11.5%). OPMD that showed the highest malignant transformation was erythroplakia (five of 12 cases (41%)), followed by lichen planus (two of five cases (40%)), leukoplakia (six of 21 cases (28%)), and OSMF (one of five cases (20%)). The erythroplakia which showed the highest malignant transformation was present for a longer duration (11.30±1.55 months in group 1, 18.40±0.00 months in group 2) (Table 1).

**Table 1:** Descriptive analysis *p-value <0.05: Significant. NS: non-significant; SLT: smokeless tobacco.

Variable	Category	Group 1 (progress to normalcy, n=29)	Group 2 (progress to malignancy, n=14)	p-Value
Gender	Male	16 (37.2%)	8 (18.6%)	0.923 (NS)
	Female	13 (30.2%)	6 (13.9%)	
Age (years)		33.65±3.75	34.64±5.82	0.523 (NS)
Type of habit	Smoking	1 (2.3%)	1 (2.3%)	0.031*
	Smoking+SLT	11 (25.5%)	3 (6.9%)	
	SLT	10 (23.2%)	0 (0%)	
	SLT+Alcohol	6 (13.9%)	3 (6.9%)	
	SLT+Smoking+Alcohol	1 (2.3%)	7 (16.2%)	
Duration of habit (years)	SLT	3.62±3.04	4.64±2.84	0.298 (NS)
	Smoking	2.00±2.44	4.35±2.79	0.007*
	Alcohol	3.10±2.89	5.01±2.14	0.034*
Site of lesion	Buccal mucosa	17 (39.5%)	5 (11.6%)	0.429 (NS)
	Palate	4 (9%)	2 (4.6%)	
	Floor of mouth	3 (6.9%)	6 (13.9%)	
	Gingiva	5 (11.6%)	1 (2.3%)	
Side	Left	17 (39.5%)	3 (6.9%)	0.022*
	Right	12 (27.9%)	11 (25.5%)	
Type of premalignant disorder	Leukoplakia	15 (34.8%)	6 (13.9%)	0.779 (NS)
	Erythroplakia	7 (16.2%)	5 (11.6%)	
	Oral submucous fibrosis	4 (9.3%)	1 (2.3%)	
	Lichen planus	3 (6.9%)	2 (4.6%)	
Duration of disorder (months)	Leukoplakia	8.50±1.23	13.20±2.34	0.001*
	Erythroplakia	11.30±1.55	18.40±0.00	0.001*
	Oral submucous fibrosis	5.60±1.25	7.20±0.68	0.001*
	Lichen planus	8.60±1.23	15.50±2.50	0.001*

Statistically significant differences were observed in the total neutrophil count (p<0.0001), lymphocyte count (p<0.001), and NLR (p<0.001) values between the groups. There was a high neutrophil count in patients with premalignancy (6.10±0.61) and malignant conditions (11.78±1.31) compared to normal (5.31±0.71) and a subsequent decrease in the total lymphocyte count in malignancy (2.42±0.51) and premalignancy (2.42±0.51) compared to normal (3.34±0.55). There was a decrease in the oral neutrophil count and a subsequent increase in the oral lymphocyte count, thus reducing the NLR values in patients who showed complete remission during the year of follow-up in group 1 at T1 when compared to T0. NLR values were the lowest in patients who had completely recovered (1.63±0.36), followed by an increase in NLR values in OPMDs (2.08±0.56), and the highest NLR values were observed in patients with malignancy (5.03±1.09) (Table 2).

**Table 2:** Paired t-test to compare NLR values, neutrophils and lymphocytes in both groups *p-value <0.05: Significant. T0: baseline; T1: one-year follow-up.

NLR values	N	T0 (mean±SD)	T1 (mean±SD)	t-Statistic	p-Value
Group 1	29	2.08±0.56	1.63±0.36	-4.23	0.001*
Group 2	14	2.38±1.37	5.03±1.09	5.344	0.001*
Neutrophil cells (10^6^)					
Group 1	29	6.10±0.61	5.31±0.71	-7.63	0.0001*
Group 2	14	6.57±0.85	11.78±1.31	13.70	0.0001*
Lymphocyte cells (10^6^)					
Group 1	29	3.10±0.72	3.34±0.55	1.75	0.081
Group 2	14	3.14±0.86	2.42±0.51	-2.68	0.018*

**Figure 3:**
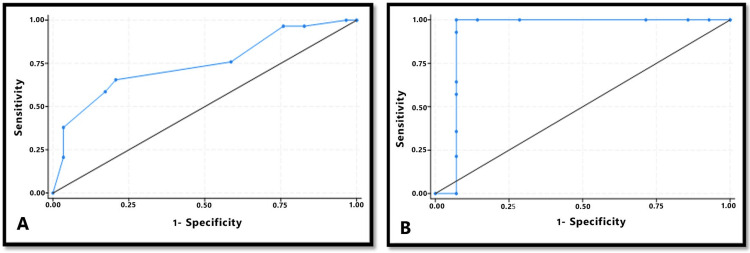
ROC curves for NLR values A. Cutoff value for predicting premalignancy in normal patients was ≥2.0 at 0.41 Youden index. Sensitivity is 82%, specificity is 58%, and area under the curve (AUC) is 0.745. B. The cutoff value for predicting malignancy in patients with OPMDs was ≥4.0 at 0.84 Youden index. Sensitivity and specificity are 92% and AUC is 0.928.

ROC curves revealed that NLR values to predict premalignancy in a normal patient with a history of tobacco or alcohol consumption showed 82% sensitivity and 58% specificity, with a cutoff value of more than or equal to 2. NLR values to predict malignancy in a patient with OPMD and a history of tobacco or alcohol consumption showed 92% sensitivity and specificity, with a cutoff value of more than or equal to 4. This shows that NLR values can be used as reliable predictors of malignancy in patients with OPMDs, whereas NLR values are less reliable in predicting premalignancy in normal patients because of their lower specificity (Figure 3).

## Discussion

OSCC accounts for approximately 90% of all oral malignancies, making it the sixth most prevalent globally [1]. Recent studies on the immune system have yielded valuable information regarding the potential influence of the host immune response on the advancement and growth of tumors. As a result, the importance of immunological biomarkers in therapeutic interventions and prognostic evaluations of oral cancer has been emphasized [4]. Approximately one-third of OSCC cases occur in patients with OPMDs [3]. Numerous studies have focused on the significance of NLR as a predictive biomarker for oral cancer [7-10], which detects circulating polymorphonuclear leukocytes (cPMNs). Neutrophilia and elevated NLR have been found to be correlated with an unfavorable prognosis in OSCC [10]. The potential impact of oral polymorphonuclear leukocytes (oPMNs) in the proximity of the tumor site on tumor development cannot be overlooked, thus emphasizing the significance of evaluating their functionalities as valuable diagnostic instruments. Nevertheless, the existing literature on oPMNs in the context of oral cancer is limited in scope, and no study has assessed NLR in the salivary samples of patients with OPMDs. Thus, the present study was conducted to assess NLR in OPMDs and to determine a cutoff value for NLR in patients who developed OSCC during the one-year follow-up period.

The role of the tumor microenvironment in tumor progression has been well established [18]. Inflammation is a crucial component of the microenvironment, wherein cells, such as neutrophils and lymphocytes, contribute significantly to tumor progression. Neutrophils are a subset of inflammatory cells that secrete various molecules, including vascular endothelial growth factor (VEGF), chemokines, and proteases, which facilitate angiogenesis, thereby establishing a conducive tumor microenvironment that promotes the development and progression of tumors. Neutrophilia encircling cancerous lesions can hinder the immune response. In comparison, the quantity of lymphocytes present within the tumor microenvironment can affect the functionality of natural killer cells. Moreover, an increase in the number of lymphocytes results in a decrease in the secretion of cytokines, which can trigger apoptosis in tumor cells. Hence, an increase in NLR has been noted to be indicative of a negative predictor of OSCC [7,12].

The present study revealed that most OPMDs were found in males, compared to females. Both alcohol consumption and tobacco use have been linked to the development of cancer. These findings were in agreement with previous studies [2,19]. Erythroplakia showed the highest malignant transformation rate, which was in agreement with previous studies [2,3]. A possible reason for this phenomenon could be attributed to the fact that erythroplakia, as a red lesion of the oral cavity, has the potential to enhance the dissemination of cancerous cells and thereby show a propensity for malignant transformation. Therefore, individuals often do not seek medical attention because of their asymptomatic nature or lack of pain associated with this condition. As a result, delayed identification and treatment of erythroplakia may allow it to progress to a more advanced stage, thereby elevating the risk of malignancy. In our study, erythroplastic lesions were present for the longest duration (18.4 months), compared to other premalignant lesions [3]. OPMDs present on the floor of the mouth showed the highest malignant transformation, which is in agreement with previous studies [2,3]. A possible explanation for this phenomenon could be attributed to the fact that the floor of the mouth has a non-keratinized squamous epithelium, which is thin and has a well-supplied network of blood vessels. This enhanced blood circulation facilitates the rapid proliferation of cancerous cells, whereas the thinner epithelium acts as a less formidable obstacle to the dissemination of malignant cells into the underlying tissues [20].

The present study found that NLR was higher in patients who developed OSCC than in those who did not develop oral cancer. This was in accordance with the results of previous studies [10,11]. The mean NLR value in patients who developed OSCC was 5.03±1.09, compared to 1.63±0.36 in patients who showed complete remission during an average follow-up period of one year. Though previous studies have reported a similar finding with an increase in NLR values in OSCC, compared to normal individuals, however, they reported lower values of 2.7-2.84 in OSCC patients [21,22]. This difference in NLR values could be due to the fact that they estimated NLR values in the blood samples of the patients, and in the present study it was estimated in the salivary samples. It has been well documented in a previous study that oPMNs produce high reactive oxygen species (ROS) compared to cPMNs [23]. Also, the difference in the methodology could have resulted in the disparities. Initial tumor growth relies on an increase in cellular proliferation and a decline in cellular mortality. The neutrophil count can function as both a promoter and a destroyer of tumors, contingent upon their state of differentiation and the influence of TGF‐beta. Neutrophil subsets can suppress T-cell proliferation through the involvement of integrin Mac-1 and hydrogen peroxide. T lymphocytes primarily inhibit the proliferation and metastasis of tumor cells through cytotoxic cell death and cytokine production, respectively. Lymphopenia can result in a reduction in the body's immune response. NLR typically remains stable in the presence of various physiological, pathological, and physical factors, although alterations in neutrophil and lymphocytic counts can occur. Consequently, a higher NLR is indicative of a poorer cancer prognosis [24].

An increased NLR has the potential to induce neutrophilia owing to the presence of tumor granulocyte colony-stimulating factor, which in turn can expedite tumor progression and augment the concentration of cytokines in the plasma, such as interleukin-6 and tumor necrosis factor-α. In contrast, lymphopenia is associated with disease severity and the ability of tumor cells to evade immune surveillance through the infiltration of lymphocytes into the tumor [25]. Neutrophilia and the subsequent lymphopenia may exert suppressive effects on lymphokine-activated killer cells, potentially leading to impaired functionality. This phenomenon could potentially contribute to the decline in survival rates among individuals with cancer. A compromised immune response resulting from lymphopenia may underlie the reduced survival observed in patients [26].

The NLR cutoff value found in our study was different from that in the previous studies [9-11], which could be because all previous studies calculated NLR in the blood samples of patients, and in the present study, salivary samples were used for estimation of NLR. Blood reflects systemic inflammation throughout the body, whereas saliva may represent localized inflammation within the oral cavity. Cancer in the oral cavity or nearby regions can affect the composition of immune cells in saliva more directly than in the bloodstream. Saliva reflects real-time changes in the oral cavity, and alterations in NLR could potentially be detected earlier or more sensitively than systemic changes reflected in the blood. The oral cavity has its own unique microenvironment, and changes in the immune cell composition of saliva may be more pronounced in response to local conditions, including the presence of oral cancers [27]. Therefore, the estimation of NLR in saliva is more reliable than the estimation of NLR in blood samples, as used in previous studies.

In the current study, increased NLR values were noted in individuals with OPMDs at the initial stage, as opposed to NLR values acquired after complete remission during the one-year follow-up period. This may be because smoking and tobacco consumption lead to the production of ROS, which reduce the responses of the host immune system and affect the viability and phagocytic activity of oral neutrophils. Neutrophilia and lymphopenia are physiological responses of the immune system to inflammation associated with OPMD, such as lichen planus, which causes neutrophilia in the saliva of patients [28,29]. Moreover, saliva loses its antioxidant capacity in the presence of chronic smoking, and the production of free radicals such as ROS and reactive nitrogen species such as nitrosamines (RNS) released from tobacco products show synergistic effects, leading to a reduced survival rate of oral lymphocytes, thus reducing its count [29]. Lymphopenia among patients with OPMDs as a consequence of their habits was greater than that observed in the oral salivary samples. This led to an increase in the NLR values in OPMDs. In the present study, all the patients were followed regularly at three-month intervals for one year, which was in accordance with the consensus guidelines on the management of OPMDs [16]. The mean NLR value in OPMDs was 2.08±0.56, compared to 1.63±0.36 in patients who showed complete remission. The cutoff NLR value between normal and OPMD was 2 (sensitivity 82%, specificity 58%, and AUC 0.745). The cutoff NLR values between OPMD and malignancy was 4 (sensitivity 92%, specificity 92%, and AUC 0.928). This finding was in agreement with a previous study by Singh et al. in 2021 [14]. However, they found higher specificity for cutoff NLR values between normal and OPMD. This might be due to differences in methodology and estimation of NLR values in the blood samples of the patients.

This study has several notable strengths, one of which is its prospective design. This study focused on the salivary samples obtained from patients diagnosed with premalignant oral disorders. These patients were administered standard medicinal treatments and motivated to cease harmful habits. Subsequently, the patients were monitored over the course of one year, at three-month intervals. The investigators also compared the baseline NLR values between patients who had fully recovered and those who had developed malignancy. It is important to note that no previous studies have been conducted on this topic. The clinical significance of this study lies in the high chance of malignant transformation in patients with OPMDs with salivary NLR values ≥4. Such individuals should be subjected to more frequent monitoring for signs of malignant transformation, particularly if they present with erythroplastic lesions affecting the floor of the mouth and have a history of alcohol or tobacco consumption.

Limitations

The primary limitation of this study was the exclusive inclusion of patients with OPMDs with mild dysplasia. Conventional medicinal treatments can only be administered in cases of mild dysplasia, and cases with moderate or severe dysplasia usually require surgical treatment. Therefore, only patients with mild dysplasia were included in this study. The study did not include other OPMDs of the oral cavity, such as erythroleukoplakia, smokers’ palates, actinic cheilitis, or xeroderma pigmentosum. The authors incorporated the most prevalent OPMDs into their study. Furthermore, this study did not assess the stages of OSCC, which is beyond the scope of this study. The present study evaluated the NLR values in the salivary samples and did not correlate these values with those from the blood samples of the patients. Consequently, future prospective studies should be conducted to ascertain NLR values in salivary samples, to correlate these values with values from blood samples of the patients and to validate the results of the present study.

## Conclusions

To the best of our knowledge, this investigation embodies a ground-breaking endeavor to investigate the values of NLR in saliva samples collected from individuals. The noninvasive, painless, sensitive, and easily obtainable characteristics of saliva make it a viable option for cost-effective screening, prompt diagnosis, efficient monitoring, and post-treatment surveillance of patients with precancerous and cancerous oral conditions. The NLR might be a potential independent prognostic indicator in patients with oral cancer. NLR values equal to or greater than 4 in patients with OPMDs exhibit a substantial likelihood of malignant progression and hence necessitate careful monitoring for definitive intervention. NLR values showed an increase in premalignant and malignant conditions. Elevated NLR values are associated with unfavorable prognosis.
